# Supplementary material for: Decreased risk of Parkinson’s disease in diabetic patients with thiazolidinediones therapy: An exploratory meta-analysis
Source: PLoS One. 2019 Oct 22;14(10):e0224236. doi: 10.1371/journal.pone.0224236 (PMC6804998; doi:10.1371/journal.pone.0224236)
Supplement: S1 File — (DOC) [file pone.0224236.s001.doc]

**S1  File.  Study  Protocol**

**Decreased risk of Parkinson’s disease in diabetic patients with thiazolidinediones therapy: An exploratory meta-analysis**

***Background***

Parkinson’s disease (PD) is a prevalent and complex neurodegenerative disorder with prominent loss of dopaminergic neurons in the substantia nigra. Rest tremor, rigidity, bradykinesia, and postural instability are the four classical motor symptoms [1]. PD is the second most common neurodegenerative disease. The annual incidence is 14/100 000 of the total population in Europe and the Americas, while the rate increased to 160/100 000 in people aged over 65 years. The incidence is similar in Asia [2]. Thiazolidinediones (TZDs), a class of peroxisome proliferator-activated receptor-gamma (PPAR-γ) agonists, can improve insulin sensitivity and lower blood glucose level for patients with type 2 diabetes [3].

Recently, accumulating experiments indicated that TZDs could exert anti-inflammatory and neuroprotective effects in a series of PD animal models [4-7]. Nevertheless, one randomized controlled trial (RCT) indicated that pioglitazone is unlikely to be able to slow the disease progression in early PD patients without diabetes [8]. To date, several retrospective observational cohort studies have assessed the association between the TZDs use and the incidence of PD in diabetic patients, but with controversial results.

Thererfore, we performed this meta-analysis to evaluate the efficacy of TZDs in reducing PD risk among diabetic patients.

***Objective***

The purpose of this meta-analysis is to explore the association between TZDs use and the incidence of PD in diabetic patients.

***Methods***

**Search strategy**

Two investigators will independently search The Cochrane Central Register of Controlled Trials (CENTRAL), PubMed, Web of Science and Embase for eligible articles before 31 December, 2018. And we will perform this meta-analysis according  to  the  recommendations  of  the Preferred  Reporting Items  for  Systematic  Reviews and Meta-analysis (PRISMA).

**Incusion and exclusion criteria**

The inclusion criteria should be:

(1) studies that investigated both TZDs use and incidence of PD among diabetic patients;

(2) comparison of TZDs with non-TZD treatments;

(3) the diagnosis of PD as an outcome.

The exclusion criteria should be:

(1) case reports, comments, reviews, editorials and clinical guidelines;

(2) studies that did not provide valid data, such as the diagnosis of PD was not the outcome or the data was incomplete.

**Data extraction**

Two investigators will independently rate the quality of retrieved studies and extract the relevant data from each study using a detailed form. We will extract the data from each study including first author’s name, publication year, location, TZDs type, sample size, follow-up duration, definition of PD, the incidence of PD.

**Risk of bias assessment**

The  quality  of  cohort  studies  will  be  appraised  according  to the Risk Of Bias In Non-randomized Studies-of Interventions (ROBINS-I tool). If necessary, disagreements will be solved by consensus or through discussion with a third reviewer.

**Strategy for data synthesis**

We will manage data using the Review Manager 5.3 software (The Nordic Cochrane Centre, The Cochrane Collaboration, Copenhagen) and set odds ratio (OR) at a 95% confidence interval (95% CI) to assess the association between TZDs use and the incidence of PD in diabetic patients. Sensitivity analysis will be performed by omitting one study per iteration. Subgroup analysis will be conducted by using TZDs type, ethnicity, and follow-up duration as stratifying variables.

***References***

[1]. Kalia LV, Lang AE. Parkinson's disease. The Lancet. 2015; 386: 896-912.

[2]. Ascherio A, Schwarzschild MA. The epidemiology of Parkinson's disease: risk factors and prevention. The Lancet Neurol. 2016; 15: 1257-1272.

[4]. Bonato JM, Bassani TB, Milani H, Vital MABF, de Oliveira RMW. Pioglitazone reduces mortality, prevents depressive-like behavior, and impacts hippocampal neurogenesis in the 6-OHDA model of Parkinson's disease in rats. Exp Neurol. 2018; 300: 188-200.

[5]. Ridder DA, Schwaninger M. In search of the neuroprotective mechanism of thiazolidinediones in Parkinson's disease. Exp Neurol. 2012; 238: 133-137.

[6]. Breidert T, Callebert J, Heneka MT, Landreth G, Launay JM, Hirsch EC. Protective action of the peroxisome proliferator-activated receptor-gamma agonist pioglitazone in a mouse model of Parkinson's disease. J Neurochem. 2002; 82: 615-624.

[7]. Pinto M, Nissanka N, Peralta S, Brambilla R, Diaz F, Moraes CT. Pioglitazone ameliorates the phenotype of a novel Parkinson's disease mouse model by reducing neuroinflammation. Mol Neurodegener. 2016; 11: 25.

[8]. Simuni T, Kieburtz K, Tilley B, Elm JJ, Ravina B, Babcock D, et al. Pioglitazone in early Parkinson's disease: a phase 2, multicentre, double-blind, randomised trial. The Lancet Neurol. 2015; 14: 795-803.
