# Supplementary material for: Decreased risk of Parkinson’s disease in diabetic patients with thiazolidinediones therapy: An exploratory meta-analysis
Source: PLoS One. 2019 Oct 22;14(10):e0224236. doi: 10.1371/journal.pone.0224236 (PMC6804998; doi:10.1371/journal.pone.0224236)
Supplement: S2 File — (DOC) [file pone.0224236.s002.doc]

**S2  File.  Search  strategy**

Relevant studies, published before 31 December 2018, were identified through the Cochrane Central Register of Controlled Trials (CENTRAL), PubMed, Web of Science and Embase databases. Electronic searches were supplemented  by scanning references of reviews and included articles to identify additional relevant studies and by correspondence with the study authors when primary outcome data was uncomplete. The compuer-based searches combined search terms related to Parkinson disease and thiazolidinediones. Below is the detailed search strategy for per database.

**the Cochrane Central Register of Controlled Trials (CENTRAL)**

(parkinson’s disease OR parkinson disease OR PD OR parkinsonism OR paralysis agitans) AND (Glitazone OR Glitazones OR Thiazolidinediones OR Thiazolidinedione OR Pioglitazone OR Rosiglitazone) in Record Title

**PubMed**

(parkinson* disease[Title] OR PD[Title] OR parkinsonism[Title] OR paralysis agitans[Title]) AND (Glitazone[Title] OR Glitazones[Title] OR Thiazolidinediones[Title] OR Thiazolidinedione[Title] OR Pioglitazone[Title] OR Rosiglitazone[Title])

**Web of Science**

TI=((parkinson’s disease OR parkinson disease OR PD OR parkinsonism OR paralysis agitans) AND (Glitazone* OR Thiazolidinedione* OR Pioglitazone OR Rosiglitazone))

**Embase**

('(parkinson*':ti OR 'pd':ti OR 'parkinsonism':ti OR 'paralysis agitans)':ti) AND ('(glitazone*':ti OR 'thiazolidinedione*':ti OR 'pioglitazone':ti OR 'rosiglitazone)':ti)
